# Multi-omics profiling implicates gut microbiota-sphingolipid interplay in the neuroprotective effects of semaglutide on diabetic cognitive impairment

**DOI:** 10.3389/fmicb.2026.1705784

**Published:** 2026-03-26

**Authors:** Liqin Qi, Huimin Kang, Xiaofen Li, Lijing Wang, Yinchen Lin, Menglan Zhan, Feihui Zeng, Zhiwen Xiao, Xiaoying Liu, Zhou Chen, Libin Liu

**Affiliations:** 1 Department of Endocrinology, Fujian Institute of Endocrinology, Fujian Medical University Union Hospital, Fuzhou, Fujian, China; 2 Department of Pediatrics, Fujian Medical University Union Hospital, Fuzhou, Fujian, China; 3 Department of Breast Surgery, Clinical Oncology School of Fujian Medical University, Fujian Cancer Hospital (Fujian Branch of Fudan University Shanghai Cancer Center), Fuzhou, Fujian, China; 4 Department of Pharmacology, College of Pharmacy, Fujian Medical University, Fuzhou, Fujian, China

**Keywords:** diabetic encephalopathy, semaglutide, gut microbiota, sphingolipid metabolism, bile acids

## Abstract

**Background:**

The gut microbiome is a critical regulator of host health, but how it mediates the therapeutic effects of drugs targeting neurodegenerative diseases like diabetic cognitive impairment (DCI) is unclear. Here, we investigated whether the neuroprotective effects of the GLP-1 agonist semaglutide (SE) are linked to its modulation of the gut-brain axis.

**Methods:**

We used an integrative multi-omics approach in a mouse model of DCI. We combined fecal shotgun metagenomics and targeted bile acid profiling with cerebral proteomics and metabolomics to characterize the gut-brain crosstalk following a 12-week SE treatment. Animal behavior, neuronal survival and synaptic integrity were assessed to confirm therapeutic efficacy.

**Results:**

SE treatment reversed cognitive deficits, rescued hippocampal neuronal loss, and restored synaptic integrity in diabetic mice. At the ecosystem level, metagenomics revealed that SE treatment profoundly remodeled the gut microbiota, enhancing microbial α-diversity, enriched beneficial genera (*Bacteroides*, *Barnesiella*), and depleted the pro-inflammatory genus *Desulfovibrio*. This microbial shift was associated with normalized fecal and cerebral bile acid profiles. Mechanistically, our analysis implicated a dysregulated sphingolipid pathway in the DCI brain, characterized by the upregulation of the transporter ATP-binding cassette transporter A2 (ABCA2) and the enzymes sphingosine-1-phosphate phosphatase 1 (SGPP1) and ceramide synthase 2 (CERS2). SE treatment dynamically modulated this pathway: it downregulated ABCA2 in a potentially weight-independent manner and SGPP1 in a weight-dependent fashion, linked to the normalization of cerebral bile acid profiles. In contrast, CERS2, a robust marker of disease severity, was not altered by SE.

**Conclusion:**

Our study uncovers a novel “gut microbiota–bile acid–sphingolipid” axis in DCI and suggests that SE acts via a dual mechanism. It drives a weight-dependent restoration of the gut-brain axis, normalizing microbial and bile acid profiles to regulate SGPP1, while also exerting weight-independent effects, potentially through direct modulation of targets like ABCA2. This work highlights the gut microbiome as a key component in the therapeutic action of SE and reveals the multifaceted nature of its neuroprotective effects.

## 1 Introduction

Diabetic cognitive impairment (DCI), a debilitating neurological complication of diabetes mellitus (DM), is characterized by progressive cognitive decline, chronic neuroinflammation, and irreversible brain structural remodeling (McCrimmon et al., 2012; Siehler et al., 2021; Simó et al., 2017). With the global diabetes pandemic affecting over 537 million individuals, DCI has emerged as a major public health challenge demanding effective therapeutic strategies (Magliano and Boyko, 2021). Semaglutide (SE), a long-acting glucagon-like peptide-1 (GLP-1) receptor agonist, has shown significant promise in mitigating cognitive dysfunction (Mahapatra et al., 2022; Zhang et al., 2019). Preclinical studies have revealed its potent neuroprotective properties, which are attributed to its ability to suppress neuroinflammation (Wang et al., 2021), enhance cerebral glucose metabolism (Wang et al., 2023), modulate autophagy (Elbadawy et al., 2025), and exert antioxidant effects (Yang et al., 2025). Despite compelling evidence of its therapeutic potential in DCI, a comprehensive understanding of its underlying mechanisms, particularly regarding the interplay between central and peripheral pathways, is still lacking.

Two interconnected pathological axes are increasingly implicated in DCI: central sphingolipid metabolism and the gut-brain axis. Within the brain, insulin resistance triggers the dysregulation of sphingolipid metabolism, leading to the accumulation of neurotoxic ceramides and a deficit in neuroprotective sphingosine-1-phosphate (S1P) (Chaurasia and Summers, 2021; Mei et al., 2023; Wang and Bieberich, 2018). This metabolic imbalance disrupts neuronal plasticity and promotes neurodegeneration, making sphingolipid pathways a prime therapeutic target (Kalinichenko et al., 2022). Concurrently, growing evidence highlights the gut-brain axis as a master regulator of neurometabolic homeostasis. Diabetes-induced gut dysbiosis can compromise intestinal barrier integrity, allowing pro-inflammatory molecules to trigger neuroinflammation (Bajinka et al., 2023; Li et al., 2024; Qin et al., 2012; Xu et al., 2017; Yu et al., 2019). Intriguingly, gut microbial metabolites, such as bile acids, are potent modulators of both host lipid metabolism and neuronal function. Recent studies have demonstrated that SE can significantly remodel the gut microbiota composition in metabolic diseases (de Paiva et al., 2024; Feng et al., 2024). However, whether SE leverages this gut-modulating capacity to correct the pathogenic sphingolipid profile in the diabetic brain remains an outstanding question. While our previous work established a general correlation between SE-induced gut microbiota shifts and cognitive improvements using 16S rRNA sequencing and brain transcriptomics (Qi et al., 2025), the specific molecular mediators and central pathways linking the gut to the brain were not elucidated.

Therefore, we hypothesized that the therapeutic action of SE involves a significant gut microbiota remodeling, and that this remodeling is associated with key metabolic shifts in the gut-brain axis, including bile acid and central sphingolipid pathways. To test this hypothesis, we employed an integrative multi-omics approach in a diabetic mouse model. By combining proteomics, metabolomics, targeted bile acid profiling, and metagenomics with *in vivo* and *in vitro* validation, we aimed to systematically dissect the molecular crosstalk between the gut and the brain, providing novel mechanistic insights into the neuroprotective effects of SE.

## 2 Materials and methods

### 2.1 Animal model and drug administration

Male C57BL/6 mice (8-week-old) were obtained from Beijing Huafukang Biotechnology Co. Ltd., (Certification No. SCXK[Jing]2019-0008). Mice were housed in specific pathogen-free (SPF) conditions within individually ventilated caging systems (Tecniplast, Italy) at 23 ± 1 °C, 50%–60% relative humidity, and a 12-h light/dark cycle. Animals were group-housed (2–3 mice per cage), with each cage containing animals from only one experimental group. Cages were distributed randomly across the housing racks to mitigate potential position-related environmental effects. All animals had *ad libitum* access to standard rodent chow and filtered water. After a 1-week acclimation period, mice were randomly assigned to four groups (*n* = 8 per group) using a computer-generated randomization sequence: (1) normal control (NC), (2) NC + semaglutide (NC + SE), (3) diabetic model (DM), and (4) DM + semaglutide (DM + SE). To ensure objectivity, all subsequent experiments were performed in a blinded manner. Investigators responsible for drug administration, behavioral testing, and data analysis were unaware of the group allocation of the animals until the study was complete. Cages were coded, and treatments (saline or SE) were prepared and labeled by a separate technician not involved in data collection. The NC and NC + SE groups were fed a standard chow diet. To induce diabetes, the DM and DM + SE groups were fed a high-fat diet (HFD; TP23400, TROPHIC) for 12 weeks, followed by intraperitoneal injections of streptozotocin (STZ; 40 mg/kg/day in citrate buffer, pH 4.5) for five consecutive days. Diabetes was confirmed by at least two random blood glucose (RBG) measurements ≥16.7 mmol/L from the tail vein using a glucometer (Onetouch Ultra2, Johnson & Johnson) on days 3, 5, and 7 post-STZ injection. Given the central role of chronic hyperglycemia in pathogenesis and to avoid stress-induced confounding in subsequent neurobehavioral evaluations, this study utilized RBG as the primary measure.

Following model validation, the NC + SE and DM + SE groups received daily subcutaneous injections of SE (Novo Nordisk, 202105AEP2). The treatment regimen consisted of a 6-day dose escalation (0.6, 1.2, 2.4, 4.8, 12, and 30 nmol/kg/day) to a maintenance dose of 30 nmol/kg/day for 12 weeks. The NC and DM groups received equivalent volumes of saline. Body weights and blood glucose were monitored weekly. Food intake was recorded over three consecutive days after the initiation of SE treatment. All procedures were approved by the Animal Ethics Committee of the Fujian Medical University (IACUC FJMU 2022-0807).

This animal study, including the behavioral assessments, was conducted as part of a larger experimental cohort, and the foundational phenotypic data have been reported in a related study focusing on brain transcriptomics (Qi et al., 2025). The present manuscript utilizes distinct biological samples from this cohort to perform an entirely new set of multi-omics analyses (shotgun metagenomics, cerebral proteomics, metabolomics, and bile acid profiling) aimed at investigating a novel mechanistic hypothesis.

### 2.2 Morris water maze (MWM) test

Spatial learning and memory were assessed using the MWM test (*n* = 8/group) (Infante-Garcia et al., 2017), which consisted of a 5-day acquisition phase followed by a probe trial on day 6. For the acquisition phase (Days 1–5), mice were trained to find a hidden platform in a fixed quadrant over five consecutive days (four 60-s trials per day with a 30-min inter-trial interval). Escape latency, the time taken to find the platform, was recorded as a measure of spatial learning. On day 6, a 60-s probe trial was conducted without the platform to assess long-term memory retention. Key parameters, including the number of target platform crossings and time spent in the target quadrant, were analyzed. Swimming trajectory and speed were recorded throughout all trials to monitor motor function.

### 2.3 Tissue collection and processing

After the MWM test, the mice were anesthetized with 2% pentobarbital sodium (50 mg/kg, intraperitoneally). After anesthesia, transcardial perfusion with phosphate-buffered saline was performed to clear vascular contents, followed by decapitation and rapid dissection of brain tissue. The cerebral cortex was selected for proteomic and metabolomic analyses due to its crucial role in cognitive functions affected by DCI (Grasby et al., 2020; Chen L. et al., 2024) and its sufficient tissue mass for robust multi-omics workflows. For each group, three brains were randomly selected and hemisected. The left hemispheres were immersion-fixed in 4% paraformaldehyde, whereas the right hippocampal tissues were fixed with a transmission electron microscopy (TEM)-optimized fixative for ultrastructural analysis. The remaining brain specimens were snap-frozen in liquid nitrogen and stored at −80 °C. Colon fecal samples were aseptically collected and stored at −80 °C, as they represent the distal gut microbial community critical for secondary bile acid metabolism (Li et al., 2019; Connell et al., 2024).

### 2.4 Histology and immunohistochemistry (IHC)

Paraformaldehyde-fixed brains (*n* = 3 per group) were paraffin-embedded and coronally sectioned (5 μm). For hematoxylin and eosin (H&E) staining, sections were deparaffinized, rehydrated, and stained with Mayer’s hematoxylin (Sigma, cat#H9627) and eosin Y (Sigma, cat#E4009) to assess general tissue morphology and histopathology. For neuronal quantification, Nissl staining was performed. Briefly, sections were incubated in 1% cresyl violet solution (OKA, cat# 71041284) at 37 °C for 30 min, followed by differentiation in graded ethanol solutions. The number of Nissl-positive neurons in different subfields of the hippocampus were counted in three non-overlapping fields per section by two independent investigators blinded to the experimental groups. Neurons were identified based on a clearly defined nucleus, nucleolus, and purple-blue Nissl bodies in the cytoplasm. The average neuronal density was calculated for each region. For IHC, sections underwent antigen retrieval (1 mM Tris-EDTA, pH 9.0, 100 °C, 15 min), peroxidase quenching (3% H2O2), and blocking (5% BSA). Sections were incubated overnight at 4 °C with primary antibodies against ABCA2 (1:100, Proteintech, 20681-1-AP), SGPP1 (1:100, Biorbyt, orb389350), and CERS2 (1:100, Affinity, DF13124). Staining was visualized using an HRP-conjugated secondary antibody and 3,3’-Diaminobenzidine (DAKO, K5007), followed by hematoxylin counterstaining.

### 2.5 Western blot (WB) analysis

Total protein was extracted from frozen cortical and hippocampal tissues using RIPA lysis buffer (Beyotime, cat#P0013B) containing a protease inhibitor cocktail (Beyotime cat#P1006). Protein concentration was determined using a BCA Protein Assay Kit (Thermo Fisher Scientific, United States). Equal amounts of protein (20 μg) per sample were separated by SDS-PAGE and transferred onto polyvinylidene fluoride membranes (Millipore, United States). The membranes were blocked with 5% non-fat milk in TBST for 1 h at room temperature and then incubated overnight at 4 °C with primary antibodies against ABCA2 (Proteintech, 20681-1-AP, 1:1000), SGPP1 (Biorbyt, orb389350, 1:800), CERS2 (Affinity, DF13124, 1:1000), and GAPDH (Cell Signaling Technology, #5174, 1:2000) as a loading control. After washing, membranes were incubated with HRP-conjugated secondary antibodies (Cell Signaling Technology, #7074, 1:1000) for 1 h. Protein bands were visualized using an ECL detection system (Biosharp, BL520B), and band intensities were quantified using ImageJ software.

### 2.6 TEM

Hippocampal tissues (1 mm^3^) (*n* = 3/group) were fixed in 2.5% glutaraldehyde (4 °C, 2 h), post-fixed in 1% osmium tetroxide, dehydrated in graded ethanol, and embedded in Epon 812 resin. Ultrathin sections (70 nm thick) were stained with uranyl acetate and lead citrate. Synaptic ultrastructure was imaged using TEM (HT7800; Hitachi, Tokyo, Japan) at 80 kV. For TEM analysis, parameters from multiple synapses (10 per animal) were measured, and the average value for each animal was used as a single biological replicate for statistical analysis (*n* = 3 animals/group).

### 2.7 Proteomic profiling

Total protein was extracted from cortical tissues (*n* = 3 per group). The cerebral cortex was selected for discovery proteomics due to its larger tissue mass, which ensures the high yield and quality of protein required for a robust DIA-MS workflow. Key findings were subsequently validated in both the cortex and hippocampus using targeted methods (IHC and WB). After tryptic digestion, peptides were analyzed using liquid chromatography-tandem mass spectrometry (LC-MS/MS) in a data-independent acquisition (DIA) mode. Raw data were processed using DIA-NN (v1.8.1) on the UniProt mouse database. For differential expression analysis, *p*-values were calculated using a two-tailed Student’s *t*-test and subsequently adjusted for multiple comparisons using the Benjamini-Hochberg (BH) procedure. Since no individual proteins reached the formal significance threshold (adjusted *p*-value < 0.05), we proceeded with an exploratory strategy for hypothesis generation. For this purpose, we used a combination of the nominal (uncorrected) *p*-value < 0.05 and a fold change > 1 to filter a set of candidate proteins for downstream pathway enrichment analysis. This approach allowed us to identify biologically relevant pathways for targeted experimental validation. Functional annotation was performed using the Gene Ontology and the Kyoto Encyclopedia of Genes and Genomes (KEGG) database (DAVID v6.8).

### 2.8 Cell culture and high-glucose (HG) treatment

The human neuroblastoma cell line SH-SY5Y (Cell Bank, Chinese Academy of Science) were cultured in DMEM/F12 Medium (Biosharp, China) supplemented with 10% fetal bovine serum (Gibco, United States) and 1% penicillin-streptomycin. Cells were maintained in a humidified incubator at 37 °C with 5% CO_2_. For the HG injury model, cells were seeded in 6-well plates (5 × 10^5^ cells/well). After overnight adherence, the medium was replaced with: (1) NC group: standard medium; (2) HG group: medium with 75 mM glucose; (3) HG + SE group: medium with 75 mM glucose and 200 nM semaglutide (MedChemExpress, United States). Cells were harvested after 24 h for molecular analysis.

### 2.9 RNA extraction and quantitative real-time polymerase chain reaction (qRT-PCR)

Total RNA was extracted using TRIzol Reagent (Invitrogen, United States). cDNA was synthesized from 1 μg of RNA using the PrimeScript™ RT Reagent Kit (Takara, Japan). qRT-PCR was performed on a Real-Time PCR System (Thermo Fisher Scientific, United States) using SYBR Green Master Mix (Vazyme, China). Relative mRNA expression was calculated using the 2^–ΔΔCt^ method, with GAPDH serving as the internal reference gene. The primer sequences used for amplification were as follows:

ABCA2 (Human): Forward, CCTCATCAAGACAGGGCGTT;
Reverse, TCACACTCTGGCTGCTCTTG.
SGPP1 (Human): Forward, AGCTGGGCAACGAACTCTTC;
Reverse, CATGACCAGCACCCAGATGA.
GAPDH (Human): Forward, GGTGTGAACCATGAGAAGTATGA;
Reverse, GAGTCCTTCCACGATACCAAAG.

### 2.10 Wide-targeted metabolomic profiling

Frozen cerebral cortex samples (−80 °C) (*n* = 3/group) were thawed on ice, homogenized using a grinder (30 Hz, 20 s), and extracted with 400 μL methanol-water (7:3, v/v) containing internal standards. After vortexing (5 min), the mixture was incubated on ice for 15 min and centrifuged (12,000 rpm, 4 °C, 10 min). The supernatant (300 μL) was cryopreserved at −20 °C for 30 min and recentrifuged (12,000 rpm, 4 °C, 3 min), and 200 μL aliquots were analyzed with LC-MS/MS (Waters Xevo TQ-S). Metabolite profiling was performed using the MetWare Database with total ion current normalization. Differential metabolites were identified via multivariate and univariate analyses, followed by KEGG pathway enrichment (false discovery rate, FDR < 0.05).

### 2.11 Bile acid metabolomic profiling

Samples (20 mg) (fecal sample *n* = 6/group, cerebral sample *n* = 3/group) were homogenized through ball milling and extracted with 495 μL of 80% methanol, supplemented with 5 μL of internal standard solution (10 μg/mL) for quantification. Protein precipitation was performed by incubating the extracts at −20 °C for 10 min, followed by centrifugation (12,000 rpm, 4 °C, 10 min). The supernatant was filtered through a Protein Precipitation Plate and analyzed using LC-MS.

### 2.12 Shotgun metagenomic sequencing

Fecal DNA (*n* = 3/group) was extracted using a FastPure Stool DNA Isolation Kit (Magnetic Beads, MJYH, China), and its quality was assessed for concentration, purity, and integrity prior to fragmentation (∼400 bp) using a Covaris M220. Paired-end libraries were constructed using the NEXTFLEX Rapid DNA-Seq Kit (Bio Scientific, United States) and sequenced on the Illumina NovaSeq™ X Plus platform (Shanghai Majorbio Biomedical Technology Co., Ltd). Raw data were processed through quality control, *de novo* assembly, gene prediction, non-redundant gene catalog construction, and functional annotation using the KEGG and Clusters of Orthologous Genes databases.

### 2.13 Statistical analysis

Data are presented as mean ± standard deviation. Statistical analyses were performed using GraphPad Prism 9.4.0 (GraphPad Software, CA, United States) and R software (v4.2.1). The significance thresholds were *p < 0.05, **p < 0.01, and ***p < 0.001. The specific statistical methods used are detailed below.

For all analyses, the individual mouse was treated as the statistical unit, with the exception of the metagenomic analysis. For the shotgun metagenomic sequencing, one mouse was randomly selected from each of the three replicate cages per group. Consequently, for all microbiome-related statistical comparisons, the cage was considered the independent statistical unit (*n* = 3 per group).

#### 2.13.1 Group comparisons

For comparisons among multiple groups (e.g., body weight, probe trial performance, protein/metabolite levels), data were first tested for normality using the Shapiro-Wilk test. For normally distributed data, a one-way analysis of variance (ANOVA) followed by Tukey’s *post-hoc* test was used. For non-normally distributed data, the non-parametric Kruskal-Wallis test followed by Dunn’s *post-hoc* test was employed.

#### 2.13.2 Repeated measures analysis

For data collected over time (e.g., escape latency in the MWM acquisition phase), a two-way repeated measures ANOVA was used to assess the effects of treatment, time, and their interaction, followed by Tukey’s *post-hoc* test for specific time-point comparisons.

#### 2.13.3 Multi-omics and multivariate analysis

Principal component analysis (PCA) for metabolomics/proteomics and principal coordinate analysis (PCoA) for metagenomic data were performed to visualize overall sample clustering. To statistically test for significant differences in community structure (β-diversity) or overall metabolic profiles between groups, Permutational Multivariate Analysis of Variance (PERMANOVA) was conducted using the adonis function in the R “vegan” package.

For differential abundance analysis in proteomics and metabolomics, a two-tailed Student’s *t*-test was used to calculate nominal *p*-values. For metagenomic features, the Kruskal-Wallis test was used. For the proteomic analysis, FDR correction using the Benjamini-Hochberg (BH) procedure was applied as the primary statistical standard. Since no proteins survived FDR correction at a 5% significance level, we adopted a relaxed criterion (nominal *p*-value < 0.05) for exploratory purposes to identify proteins for generating hypotheses for subsequent validation.

#### 2.13.4 Correlation and covariate analysis

Correlations between molecular/microbial features and behavioral outcomes were evaluated using the Spearman rank correlation test. To control for the confounding effect of body weight, an Analysis of Covariance (ANCOVA) was performed on key variables, with the final body weight entered as a covariate. Detailed model outputs, including the test of inter-subject effects and *post hoc* comparisons, are provided in Supplementary Table 1. To account for the multiple correlations tested throughout the study, a global Benjamini-Hochberg procedure was applied to all correlation *p*-values. As this was an exploratory study, we report both the nominal *p*-values and the adjusted *q*-values in Supplementary Data 2.

## 3 Results

### 3.1 SE ameliorates metabolic dysfunction, cognitive deficits, and hippocampal neurodegeneration in diabetic mice

**FIGURE 1:**
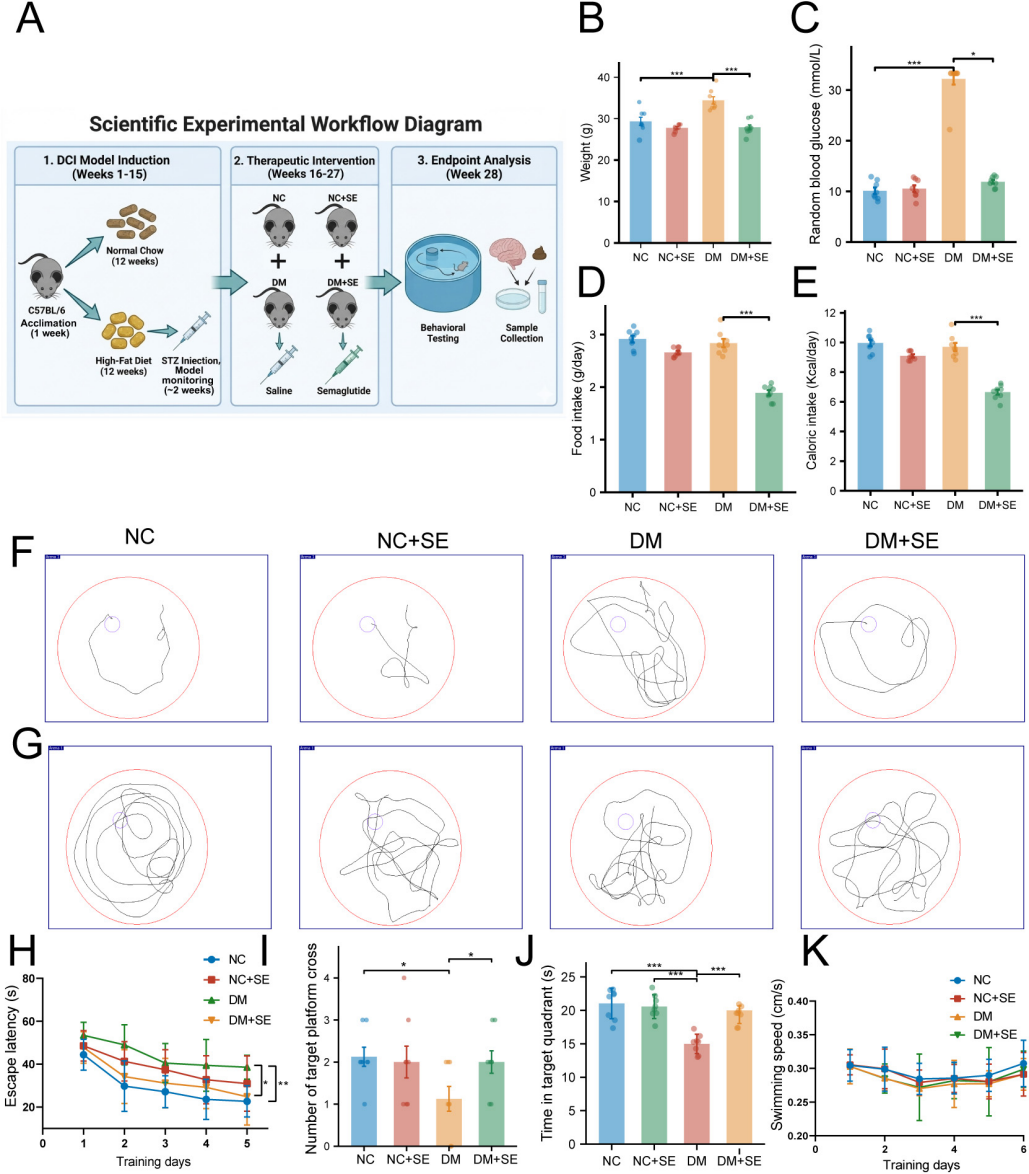
Semaglutide ameliorates systemic metabolic deficits and cognitive impairment in diabetic mice. **(A)** Schematic of the experimental design and timeline. **(B–K)** Behavioral and metabolic data establishing the diabetic cognitive impairment (DCI) model phenotype. We note that these foundational behavioral data are from the same animal cohort as reported in our previous work (Qi et al., 2025), and are presented here to provide the necessary context for the novel mechanistic data that follows. **(B)** Body weight, **(C)** random blood glucose (RBG) levels, and **(D)** food intake, and **(E)** caloric intakes in NC, NC + SE, DM, and DM + SE groups (*n* = 8/group). **(F)** Typical trajectory maps of mice during the acquisition trial phase on day 5. **(G)** Exploration trajectory maps during the probe trial on day 6. **(H)** Escape latencies across groups during the 5-day acquisition trial phase (*n* = 8/group). **(I)** Number of target platform crossings in the probe test of day 6 (*n* = 8/group). **(J)** Time spent in the target quadrant during the probe trial on day 6. **(K)** Swimming speeds across all trial days. NC, normal control group; NC + SE, normal control with semaglutide group; DM, diabetes mellitus group; DM + SE, diabetes mellitus with semaglutide group. Data are presented as mean ± SD. *p < 0.05, **p < 0.01, ***p < 0.001.

To establish a DCI model, we fed mice an HFD combined with STZ injections (Figure 1A). DM mice exhibited significant increases in body weight and RBG levels compared to NC (Figures 1B, C). A 12-week treatment with SE normalized both weight gain and hyperglycemia in DM mice (Figures 1B, C). These metabolic improvements were accompanied by a significant reduction in food and caloric intake (Figures 1D, E).

We next evaluated the impact of these metabolic changes on cognitive function using the MWM test. Notably, SE administration to healthy control mice did not significantly affect cognitive performance compared to the NC group. In the 5-day acquisition phase, DM mice showed significantly longer escape latencies than NC mice, indicating impaired spatial learning. SE treatment robustly reversed this deficit (Figures 1F, H). In the probe trial on day 6, SE-treated mice crossed the former platform location more frequently (Figures 1G, I) and spent significantly more time in the target quadrant than untreated DM mice. SE treatment rescued this memory impairment, with the performance of DM + SE mice being indistinguishable from that of control animals (Figure 1J). Importantly, swimming speeds were comparable across all groups, throughout the experiment, including on day 6, ruling out motor function as a confounding factor (Figure 1K). Collectively, these results suggest that SE ameliorate both the systemic metabolic and cognitive features of DCI in our model.

**FIGURE 2:**
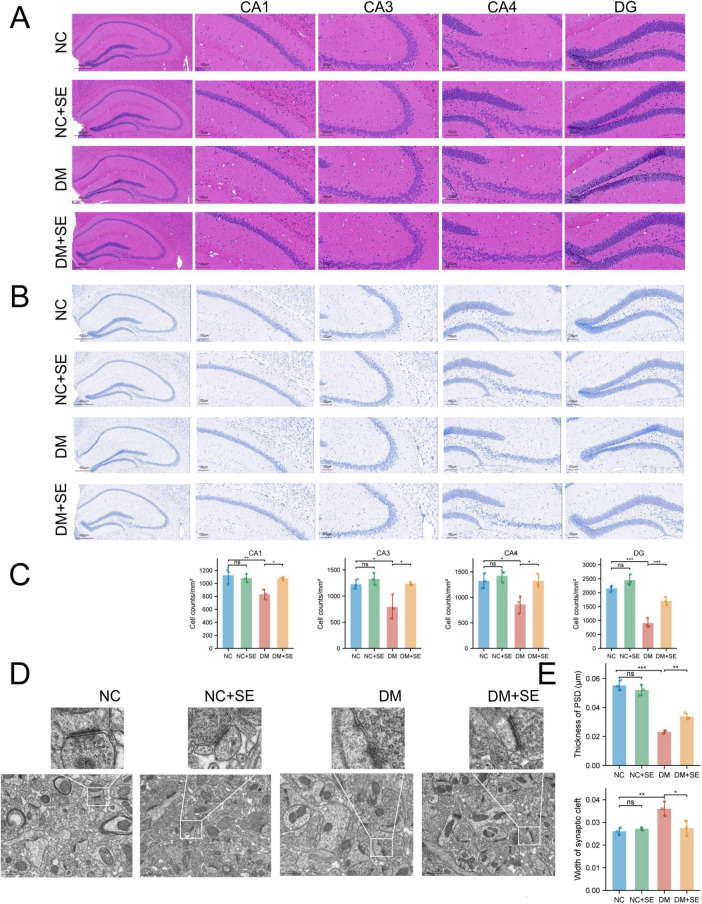
Semaglutide reverses hippocampal neurodegeneration and synaptic damage in diabetic mice. **(A)** Representative images of hematoxylin-eosin (H&E) staining showing general histopathology. Scale bar = 400 μm for low magnification images and 100 μm for high magnification images. **(B)** Representative Nissl staining images of adjacent sections used for neuronal quantification. **(C)** Quantitative analysis of Nissl-positive neuronal density in the hippocampal CA1, CA3, CA4, and dentate gyrus (DG) subfields (*n* = 3/group). **(D)** Transmission electron microscopy (TEM) images of the synaptic ultrastructure of the hippocampus. Scale bar = 1 μm. **(E)** Statistical evaluation of synaptic parameters, including postsynaptic density (PSD) thickness and synaptic cleft width (*n* = 3 animals/group). NC, normal control group; SE, normal control with semaglutide (NC + SE) group; DM, diabetes mellitus group; DM + SE, diabetes mellitus with SE group. Data are presented as mean ± SD. Statistical significance was determined by one-way ANOVA with Tukey’s *post-hoc* test. *p < 0.05, **p < 0.01, ***p < 0.001. ns, not significant.

Histopathological analysis of the hippocampus was performed to assess neurodegeneration. H&E staining revealed signs of neuronal damage, such as cell shrinkage and pyknosis, particularly in the CA1 and dentate gyrus (DG) regions (Figure 2A). To accurately quantify neuronal loss, we employed Nissl staining, a more specific marker for neurons. This exploratory analysis suggested a reduction in the density of Nissl-positive neurons in these hippocampal subfields in DM mice—a potential hallmark of neurodegeneration. Interestingly, SE treatment rescued this neuronal loss, bringing it closer to control levels (Figures 2B, C).

At the ultrastructural level, TEM imaging showed that DM mice had thinner postsynaptic densities (PSDs) and wider synaptic clefts, indicative of synaptic damage. SE treatment effectively restored both PSD thickness and synaptic cleft width to near-normal levels (Figures 2D, E). Collectively, these data suggest that SE alleviate the key metabolic, cognitive, and neuropathological features of DCI in our model.

### 3.2 Proteomics and targeted validation identify sphingolipid metabolism as a key pathway modulated by SE

**FIGURE 3:**
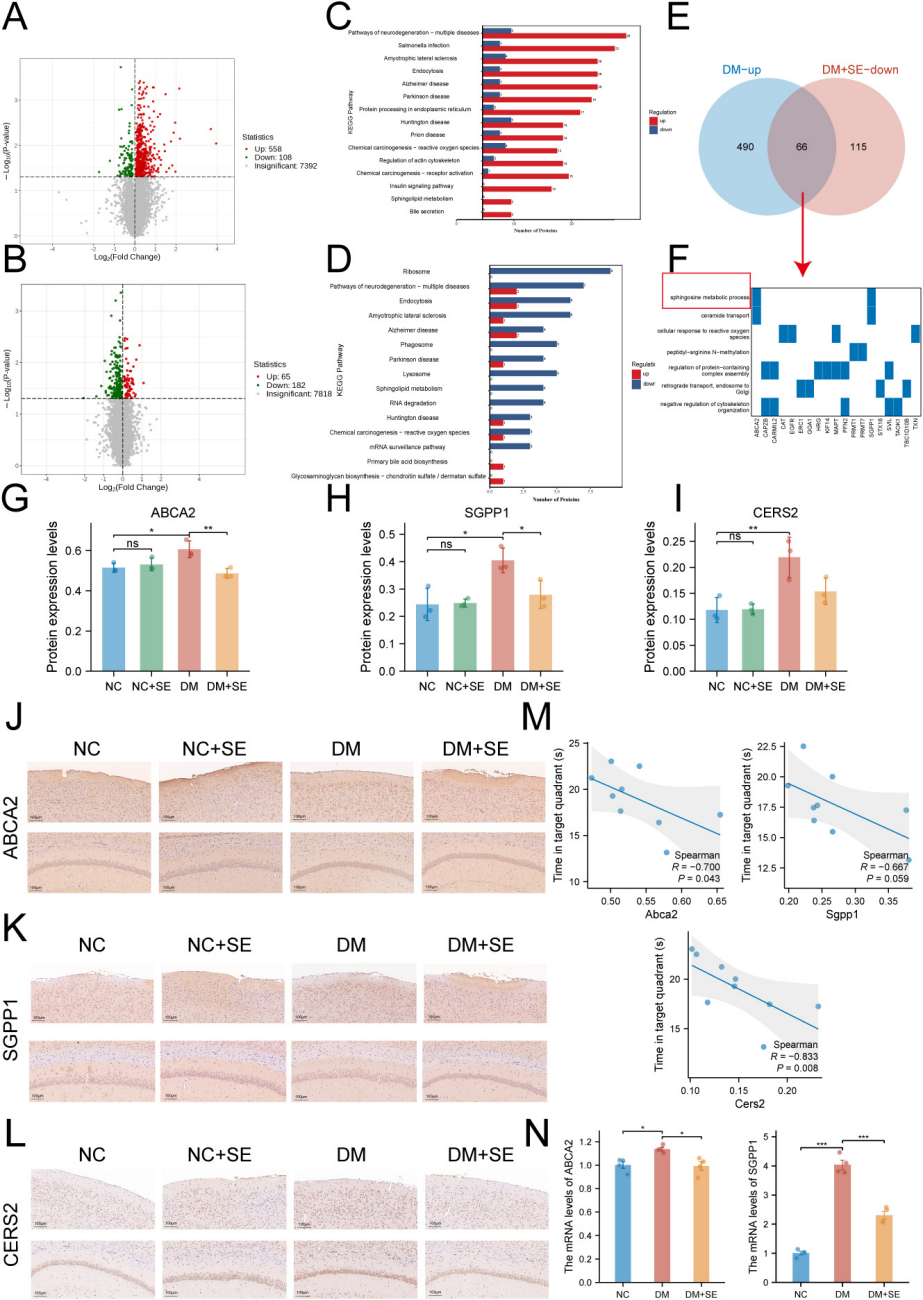
Proteomics and targeted validation identify dysregulated sphingolipid metabolism as a key therapeutic target of semaglutide in diabetic cognitive impairment (DCI). **(A,B)** Volcano plots of cortical proteins from **(A)** DM vs. NC and **(B)** DM + SE vs. DM comparisons. Dashed lines indicate the filtering criteria of uncorrected *p* < 0.05 and fold change > 1. **(C,D)** KEGG pathway enrichment analysis in NC vs. DM comparisons **(C)** and DM vs. DM + SE comparisons **(D)**. **(E)** Venn diagram identifying 66 overlapping proteins between the DM-upregulated and DM + SE-downregulated clusters. **(F)** KEGG analysis of these overlapping proteins revealed functional convergence in sphingolipid metabolism. **(G–L)** Protein expression levels **(G–I**, *n* = 3/group) and immunohistochemical staining **(J–L**, *n* = 3/group) of key sphingolipid regulators (ABCA2, SGPP1, and CERS2) in cortical and hippocampal tissues across groups. Scale bar = 100 μm. Upper: Cortex. Lower: Hippocampus. **(M**) Spearman correlation analysis between protein expression levels and memory performance (time in target quadrant). **(N)** qRT-PCR analysis of *ABCA2* and *SGPP1* mRNA in HG-treated SH-SY5Y cells with or without SE (*n* = 3 per experiment). NC, normal control group; NC + SE, normal control with semaglutide (SE) group; DM, diabetes mellitus group; DM + SE, diabetes mellitus with SE group; KEGG, The Kyoto Encyclopedia of Genes and Genomes pathway; ABCA2, ATP-binding cassette transporter A2; SGPP1, sphingosine-1-phosphate phosphatase 1; CERS2, ceramide synthase 2. Data are presented as mean ± SD. Statistical significance was determined by one-way ANOVA with Tukey’s *post-hoc* test **(G–I,N)** or Spearman correlation **(M)**. *p < 0.05, **p < 0.01, ***p < 0.001. ns, not significant.

To uncover the molecular mechanisms underlying SE’s neuroprotective effects, we first conducted an exploratory proteomic analysis of the cerebral cortex. Using a nominal *p*-value < 0.05 as a screening filter, we identified numerous candidate proteins dysregulated in DM mice and restored by SE treatment (Figures 3A, B). Although no single protein was statistically significant after FDR correction (Supplementary Data 1), a powerful biological signal emerged at the pathway level. KEGG analysis revealed that neurodegenerative pathways (e.g., Alzheimer’s disease, Huntington’s disease) were significantly enriched in DM mice, and this enrichment was markedly attenuated by SE treatment (Figures 3C, D).

To identify key therapeutic targets, we focused on the 66 proteins that were upregulated in the DM group and downregulated by SE (Figure 3E). Strikingly, functional analysis of these overlapping proteins highlighted sphingolipid metabolism as a significantly enriched pathway (Figure 3F). This finding suggests the possibility that a coordinated correction of sphingolipid dysregulation may be central to SE’s mechanism of action.

Based on this robust pathway-level hypothesis, we proceeded with targeted validation of key regulators: ABCA2, SGPP1, and CERS2. We found that the NC + SE group showed no significant differences compared to the NC group, indicating minimal effects of SE in healthy animals. Proteomic data indicated that all three were upregulated in DM mice (Figures 3G–I). Subsequent validation by IHC (Figures 3J–L) and quantitative WB (Supplementary Figures 1, 2) appeared concordant with these observations in both the cortex and hippocampus, pointing toward a potential coordinated pathological response across these cognitive brain regions.

Interestingly, SE treatment elicited distinct responses among these proteins. It significantly downregulated the expression of ABCA2 and SGPP1, but not CERS2, which remained elevated despite the treatment (Figures 3G–I). To dissect the influence of systemic metabolic changes, we performed an ANCOVA with body weight as a covariate. The therapeutic effect on ABCA2 remained statistically significant after adjusting for body weight, suggesting this effect might not be solely explained by weight loss. In contrast, the effect on SGPP1 lost its significance after the same adjustment, indicating it is more tightly coupled with the systemic metabolic improvements induced by SE.

To explore the functional relevance of these proteins, we performed an initial correlation analysis between their expression levels and memory performance (Figure 3M). We noted several trends consistent with our hypothesis. CERS2 expression showed the strongest nominal negative correlation with cognitive function (ρ = −0.833, *p* = 0.008), suggesting it may be a marker of disease severity. ABCA2 also showed a nominal negative correlation (ρ = −0.700, *p* = 0.043), while SGPP1 exhibited a similar negative trend (ρ = −0.667, *p* = 0.059). It is important to note that these correlations are exploratory in nature and did not survive a global correction for multiple comparisons (see Supplementary Data 2 for all *p*- and *q*-values).

Finally, to test for a direct effect on neuronal cells, we used an *in vitro* HG injury model. HG stimulation significantly increased *ABCA2* and *SGPP1* mRNA expression in SH-SY5Y neuroblastoma cells. Co-treatment with SE completely abrogated this increase (Figure 3N). Collectively, these multi-modal validation data suggest the sphingolipid pathway, particularly the SE-responsive enzymes ABCA2 and SGPP1, as key molecular targets implicated in SE’s neuroprotective effects in DCI.

### 3.3 Metabolomic profiling confirms SE-mediated normalization of the sphingolipid pathway

**FIGURE 4:**
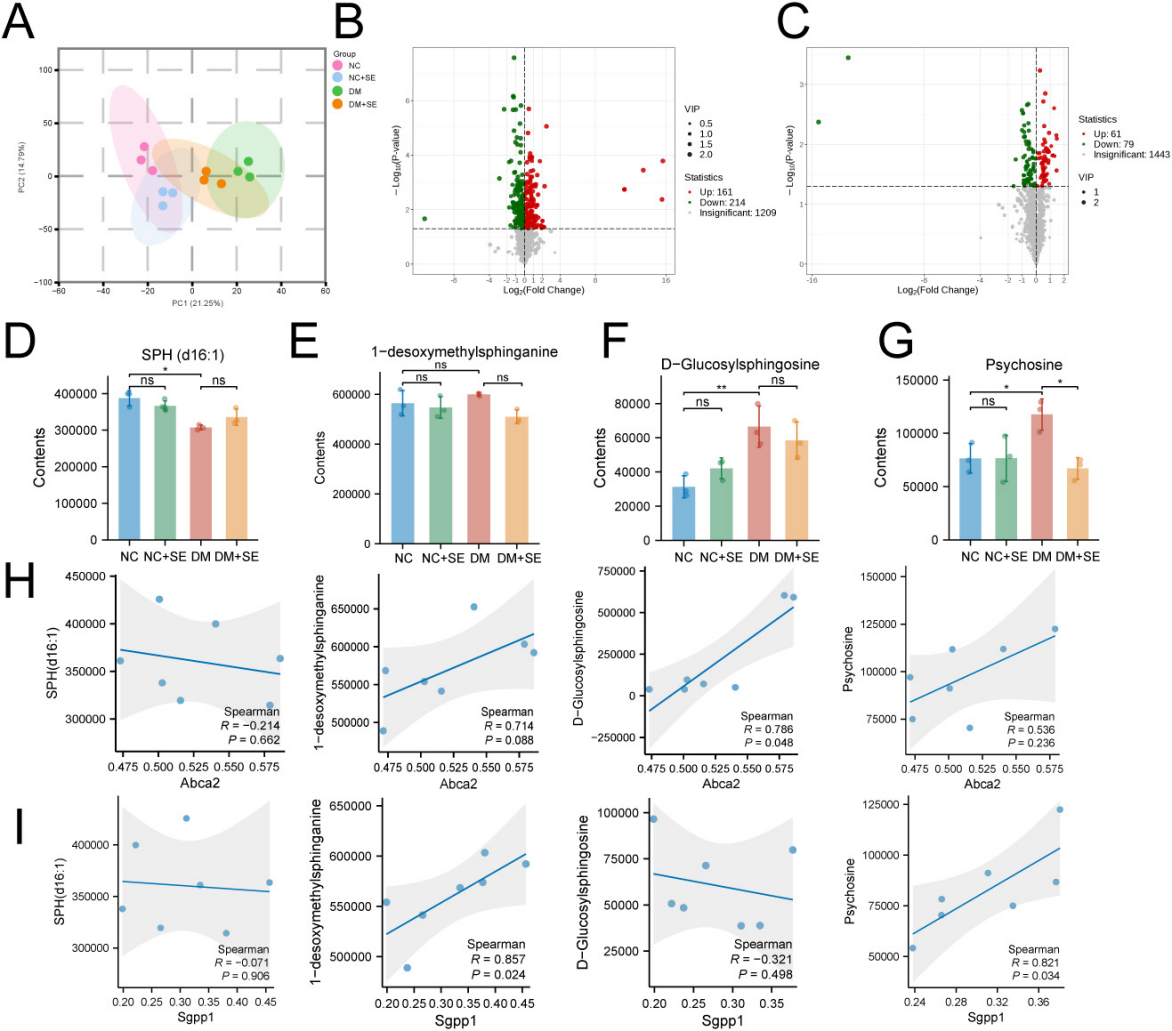
Metabolic landscape and sphingolipid network modulation by semaglutide. **(A)** Principal component analysis (PCA) of metabolomic profiles (*n* = 3/group). **(B,C)** Volcano plots of differential metabolites in panels **(B)** DM vs. NC and **(C)** DM + SE vs. DM comparisons. **(D–G)** Differential expression of sphingolipid metabolites including **(D)** SPH (d16:1), **(E)** 1-desoxymethylsphinganine, **(F)** D-glucosylsphingosine, and **(G)** psychosine levels (*n* = 3/group). **(H,I)** Spearman correlation analysis revealing significant associations between ABCA2 **(H)**/SGPP1 **(I)** expression and specific sphingolipid metabolites. NC, normal control group; NC + SE, normal control with semaglutide group; DM, Diabetes mellitus group; DM + SE, diabetes mellitus with semaglutide group; SPH (d16:1), sphingosine (d16:1); ABCA2, ATP-binding cassette transporter A2; SGPP1, sphingosine-1-phosphate phosphatase 1. Data are presented as mean ± SD. Statistical significance was determined by one-way ANOVA with Tukey’s *post-hoc* test **(D–G)** or Spearman correlation **(H,I)**. *p < 0.05, **p < 0.01. ns, not significant.

Following the proteomic findings, we performed metabolomics to determine if the enzymatic changes translated to altered metabolite levels. Principal component analysis (PCA) of the metabolomic data showed a clear separation between NC and DM groups, with the DM + SE group clustering closer to the NC group, indicating metabolic normalization (Figure 4A). Volcano plots highlighted numerous metabolites that were dysregulated in DM mice and restored by SE treatment (Figures 4B, C).

Focusing on the sphingolipid pathway, we found that levels of the neuroprotective precursor SPH (d16:1) were reduced in DM mice (Figure 4D). Conversely, levels of the neurotoxic metabolites D-glucosylsphingosine and psychosine but not 1-desoxymethylsphinganine were significantly elevated in the DM group. SE treatment effectively counteracted this pathological shift. It significantly reduced the levels of psychosine and showed a strong trend toward normalizing D-glucosylsphingosine (Figures 4E–G).

To mechanistically link these metabolite changes back to their upstream regulators, we continued our exploratory correlation analysis (Figures 4H,I). We found a strong nominal positive correlation between SGPP1 expression and both its substrate precursor, 1-desoxymethylsphinganine (ρ = 0.857, *p* = 0.024), and the neurotoxic product psychosine (ρ = 0.821, *p* = 0.034). Furthermore, ABCA2 expression showed a nominal positive correlation with the neurotoxic D-glucosylsphingosine (ρ = 0.786, *p* = 0.048). These consistent trends suggest a functional link between the expression of these proteins and the flux of the sphingolipid pathway.

### 3.4 SE normalizes pathological bile acid profiles in both the gut and the brain

Given that gut microbial shifts profoundly alter host metabolism, and that gut-derived metabolites can influence central nervous system function, we hypothesized that bile acids might be a key link in the gut-brain axis modulated by SE. We therefore profiled bile acids in both feces and the brain.

**FIGURE 5:**
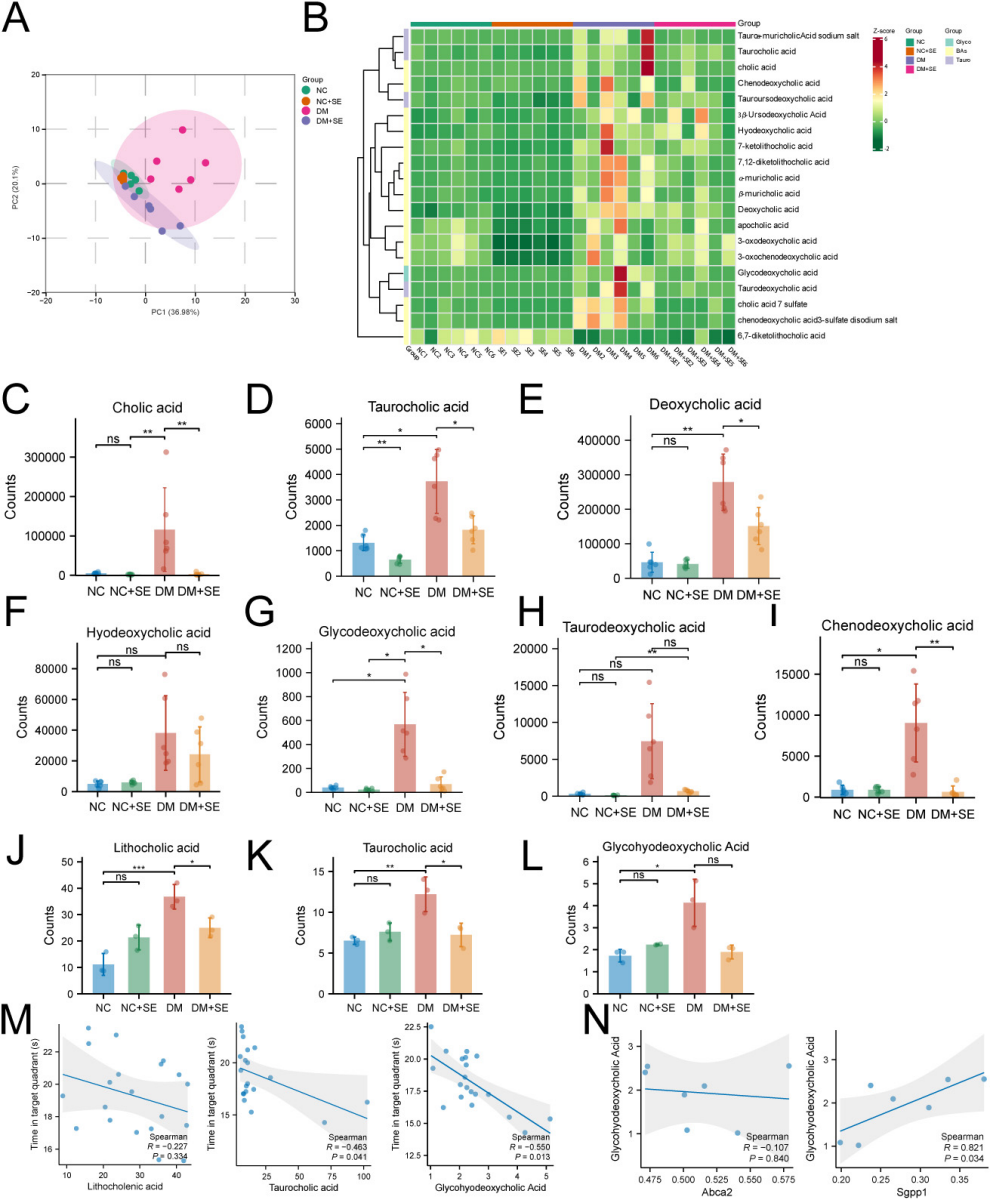
Semaglutide remodels bile acid metabolism in the gut and brain. **(A)** Principal component analysis (PCA) of fecal bile acid profiles (*n* = 6/group). **(B)** Heatmap of fecal bile acid composition. **(C–I)** Levels of representative fecal bile acids. **(J–L)** Levels of key bile acids detected in the cerebral cortex (*n* = 3/group). **(M)** Spearman correlation analysis between the levels of cerebral bile acids (LCA, TCA, GHDCA) and memory performance (time in target quadrant). **(N)** Spearman correlation between cerebral GHDCA and cortical ABCA2/SGPP1 expression. NC, normal control group; NC + SE, normal control with semaglutide group; DM, diabetes mellitus group; DM + SE, diabetes mellitus with semaglutide group; ABCA2, ATP-binding cassette transporter A2; SGPP1, sphingosine-1-phosphate phosphatase 1; BAs, bile acids. Data are presented as mean ± SD. Statistical significance was determined by one-way ANOVA with Tukey’s *post-hoc* test **(C–L)** or Spearman correlation **(M,N)**. *p < 0.05, **p < 0.01, ***p < 0.001. ns, not significant.

Fecal bile acid PCA showed distinct clustering by group, with SE treatment shifting the DM profile toward that of NC mice (Figures 5A, B). DM mice exhibited a significant increase in various fecal bile acids, including the conjugated bile acid taurocholic acid (TCA), deoxycholic acid (DCA), glycodeoxycholic acid (GDCA), and chenodeoxycholic acid (CDCA). SE treatment significantly lowered the levels of these potentially neurotoxic bile acids (Baloni et al., 2020; Ren et al., 2024; Figures 5C–I).

Crucially, this peripheral dysregulation was mirrored in the brain. Cerebral levels of lithocholic acid (LCA) and TCA were elevated in the brains of DM mice and significantly reduced by SE treatment (Figures 5J, K). In contrast, glycohyodeoxycholic acid (GHDCA), another bile acid significantly elevated in the diabetic brain, was not significantly reduced by SE, suggesting it represents a more entrenched feature of the neuropathology (Figure 5L). As an exploratory analysis, ANCOVA suggested that the elevation of these cerebral bile acids in DM mice (compared to NC) persisted after adjusting for body weight. In contrast, the therapeutic reduction of these bile acids by SE lost statistical significance after the same adjustment, indicating that this normalization is tightly coupled with SE-induced weight loss (Supplementary Table 1).

We next sought to link these cerebral bile acid changes to their functional consequences. This analysis revealed strong nominal negative correlations between memory performance and the cerebral levels of both TCA (ρ = −0.463, *p* = 0.041) and GHDCA (ρ = −0.550, *p* = 0.013), underscoring their potential pathological relevance (Figure 5M).

Strikingly, a final exploratory correlation analysis provided an insight into the gut-brain connection. We found a strong nominal positive correlation specifically between the cerebral bile acid GHDCA and the expression of SGPP1 (ρ = 0.821, *p* = 0.034), a link not observed for ABCA2 (Figure 5N). While exploratory, this specific association points toward the GHDCA-SGPP1 axis as a candidate link between gut-derived metabolic signals and central sphingolipid dysregulation in DCI.

### 3.5 Metagenomic analysis demonstrates that SE reshapes the gut microbiome and its metabolic function

**FIGURE 6:**
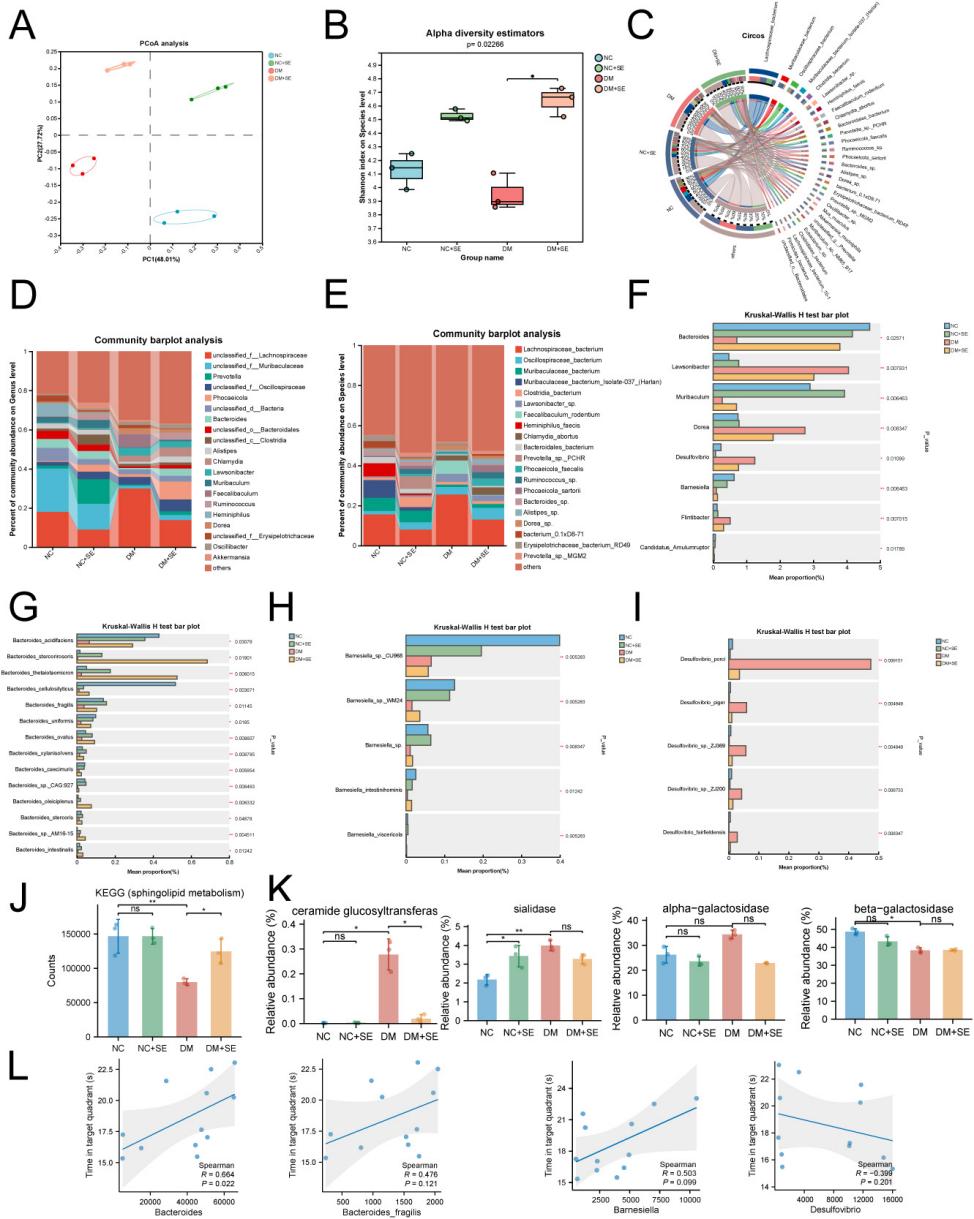
Semaglutide remodels the gut microbiota composition and functional potential. **(A)** Principal coordinate analysis (PCoA) of β-diversity (*n* = 3/group). **(B)** Shannon index for α-diversity. **(C)** Circos plot of the 30% most abundant microbial species. **(D,E)** Stacked bar plots delineating semaglutide-driven taxonomic shifts at the **(D)** genus and **(E**) species levels. **(F)** Comparative analysis of genus level differences. **(G–I)** Comparative analysis of species level differences in panels **(G)** *Bacteroides*, **(H)** *Barnesiella*, and **(I)** *Desulfovibrio*. **(J)** KEGG pathway analysis showing the abundance of microbial genes related to sphingolipid metabolism. **(K)** Enzymatic activity profiling of sphingolipid metabolism (*n* = 3/group). **(L)** Spearman correlation between the abundance of specific taxa and memory performance (time in target quadrant). NC, normal control group; NC + SE, normal control with semaglutide group; DM, diabetes mellitus group; DM + SE, diabetes mellitus with semaglutide group; KEGG, The Kyoto Encyclopedia of Genes and Genomes pathway. Data are presented as mean ± SD. Statistical significance was determined by Kruskal-Wallis test or Spearman correlation. *p < 0.05, **p < 0.01. ns, not significant.

To investigate the origin of the altered bile acid profiles, we performed shotgun metagenomic sequencing of fecal samples. Principal coordinate analysis revealed a clear separation in microbial community structure (ß-diversity) between the groups (Figure 6A). SE treatment significantly increased the α-diversity (Shannon index), which was reduced in DM mice, indicating a restoration of microbial richness (Figure 6B).

Circos visualization of the top 30% abundant species demonstrated cohort-specific microbial partitioning (Figure 6C), whereas stacked bar plots delineated SE treatment-driven shifts in dominant genera (Figure 6D) and species (Figure 6E). At the taxonomic level, DM mice showed a depletion of *Bacteroides* and *Barnesiella* (Lan et al., 2006) and an enrichment of the pro-inflammatory genus *Desulfovibrio*. SE treatment effectively reversed these changes (Figures 6F–I). Our exploratory ANCOVA showed that these restorative effects on the microbiota were no longer statistically significant after adjusting for body weight, suggesting they are associated with the systemic metabolic improvements and weight loss induced by SE (Supplementary Table 1).

Functionally, KEGG analysis showed that the genetic potential for sphingolipid metabolism within the microbiome was suppressed in DM mice and restored by SE (Figure 6J). This was associated with changes in microbial enzymes like ceramide glucosyltransferase (Figure 6K). Finally, we linked these microbial changes to host cognitive function. The abundance of the genus *Bacteroides* was found to have a nominal positive correlation with cognitive performance (ρ = 0.664, *p* = 0.022), suggesting a potential neuroprotective role for this taxon (Figure 6L). These data suggest that SE’s therapeutic action may originate, at least in part, from a remodeling of the gut microbial ecosystem, fostering the growth of beneficial taxa that are functionally linked to improved host cognition.

## 4 Discussion

It is important to contextualize our findings with respect to our recent publication in *Neurotherapeutics* (Qi et al., 2025). That initial study provided a broad, exploratory overview, linking SE’s effects to the gut-brain axis through 16S rRNA and brain transcriptomics, and pointing toward “neuroactive ligand-receptor interactions.” The present study represents a substantial and targeted advancement on that work. By employing deeper methodologies—including shotgun metagenomics, cerebral proteomics, and targeted bile acid profiling—we have proposed and provided evidence for a specific mechanistic pathway: the “gut microbiota–bile acid–spholipid” axis. The identification of sphingolipid dysregulation as a potential target and the establishment of bile acids as the specific link are the key novel contributions of this manuscript, offering a deeper mechanistic insight.

Our study further elucidates this novel axis, implicating it as a central component of SE’s neuroprotective effects in DCI. Through an integrated multi-omics approach, we demonstrate how SE-induced remodeling of the gut microbiome normalizes pathological bile acid profiles, which in turn corrects dysregulated sphingolipid metabolism in the brain. This systems-level view provides a new framework for understanding the intricate link between gut ecology and brain health in the context of metabolic disease.

A key strength of our study is the inclusion of the NC + SE control group, which establishes the context-dependent nature of SE’s action. Our data consistently show that SE exerted minimal effects on the gut microbiota, bile acid profiles, and cerebral sphingolipid metabolism in healthy, non-diabetic mice. This is a crucial finding, as it suggests that the profound benefits observed in the DM + SE group represent a targeted reversal of diabetic pathology rather than a non-specific drug effect.

At the heart of the central pathology, our proteomics and metabolomics data converged on the dysregulation of sphingolipid metabolism as a plausible key driver of DCI. We identified distinct roles for the key proteins involved. CERS2 emerged as a marker of disease severity, given its strong correlation with memory deficits, yet it was resistant to SE treatment, suggesting it may represent a more entrenched aspect of the neuropathology. In contrast, both the transporter ABCA2 (Davis, 2015) and the enzyme SGPP1 (Haughey et al., 2010; Jêśko et al., 2019; Varma et al., 2018) were dynamically modulated by SE. The upregulation of these proteins in DCI is associated with the accumulation of psychosine, a process effectively countered by SE. This multi-target engagement within a single pathway underscores the comprehensive nature of SE’s effects.

The origin of this central metabolic correction appears to lie in the profound remodeling of the gut microbiome. Our metagenomic data revealed a SE-driven shift away from a pro-inflammatory dysbiotic state—characterized by the depletion of *Bacteroides* and enrichment of *Desulfovibrio*—toward a neuroprotective microbial signature. The restoration of beneficial taxa like *Bacteroides*, which are critical for host bile acid metabolism (Chen Z. et al., 2024), likely underpins the observed normalization of bile acid profiles in both the gut and brain. This suggests that SE acts, in part, as a “prebiotic-like” agent, fostering a healthier gut ecosystem that reduces the flux of detrimental gut-derived metabolites, such as the bile acid TCA, to the brain.

A crucial insight from our study is the suggestion that SE’s effects may be mediated through at least two complementary pathways, an idea prompted by our exploratory covariate analysis. While acknowledging the limitations of using ANCOVA to infer causality for a potential mediator like body weight, the analysis provides a useful preliminary framework. On one hand, we propose an indirect “Gut-Brain Axis Restoration” pathway whose effects appear tightly coupled with weight loss. Our data show that the normalization of the gut microbiome, cerebral bile acids, and the downregulation of SGPP1 are all closely associated with SE-driven systemic metabolic improvements. The nominal correlation we identified between the cerebral bile acid GHDCA and SGPP1 expression provides a candidate molecular link for this pathway. On the other hand, SE may also utilize a pathway whose effects are less directly tied to weight loss, which we term a “Neuronal Support” pathway. The downregulation of ABCA2, which remained statistically significant after adjusting for body weight and was validated in our direct neuronal cell model, is the primary evidence for this proposed pathway. This hypothesized dual framework, where pathways closely linked to systemic metabolic health may converge with those acting more directly on neuronal targets, offers a more comprehensive perspective for understanding the potent neuroprotective efficacy of GLP-1 agonists. It highlights a testable model for future research to formally dissect through methods like mediation analysis.

Our proposal of a dual, synergistic mechanism is biologically plausible. While large peptides like SE do not readily cross the blood-brain barrier, they are known to access the brain through circumventricular organs (CVOs)—specialized regions with fenestrated capillaries (Gabery et al., 2020; Dong et al., 2022). This provides a direct anatomical route for systemically administered SE to influence central neurons. Crucially, our *in vitro* data provides direct evidence for this central action, demonstrating that SE can modulate *ABCA2* and *SGPP1* expression in neuronal cells independent of any peripheral factors.

We therefore propose an integrated model where this direct central action via CVOs works in concert with the indirect, peripherally-mediated gut-brain axis. The direct pathway may initiate a rapid, targeted response in specific neuronal circuits, while the indirect pathway—driven by a restored gut microbiome and normalized bile acid profiles—provides a sustained, systemic improvement in the metabolic and inflammatory environment. Both pathways appear to converge on the regulation of sphingolipid homeostasis, with our data identifying ABCA2 and SGPP1 as potential key molecular nodes.

This study contributes to our understanding of DCI and SE’s therapeutic action by proposing a novel, systems-level hypothesis. By integrating multiple omics layers, our work offers a preliminary, systems-level glimpse into a pathogenic axis that may transcend single-pathway studies. The findings suggest a potential therapeutic mechanism for SE that extends beyond its known GLP-1R agonism, and point toward specific targets like ABCA2, SGPP1, and Bacteroides that may warrant further investigation for future therapies.

However, several limitations should be acknowledged. Our study establishes a strong correlational framework, but definitive causal links require further validation. Future studies employing fecal microbiota transplantation (FMT), direct administration of metabolites like GHDCA, and brain-specific knockout models of *Sgpp1* and *Abca2* are needed to formally test the causal chain we have proposed. Furthermore, while our data suggest a dual mechanism, the current study was not designed to quantitatively dissect the relative contribution of the direct central and indirect peripheral pathways, a task requiring future experiments such as local CVO antagonism.

Several aspects of our study design also warrant caution. Our initial omics analyses were performed on a small sample size (e.g., *n* = 3 per group for proteomics and metagenomics), suitable for hypothesis generation but requiring confirmation in larger cohorts. Specifically for the microbiome analysis, we did not include a pair-fed control group, making it difficult to fully disentangle the direct pharmacological effects of SE on the gut microbiota from those secondary to reduced caloric intake. While we treated the cage as the statistical unit, potential cage effects cannot be entirely ruled out. Additionally, our DCI model characterization was limited to random blood glucose, and future work would benefit from including IPGTT/ITT. Finally, while we identified several strong correlational trends, none of these associations survived a stringent global correction for multiple comparisons, underscoring the exploratory nature of these findings.

**FIGURE 7:**
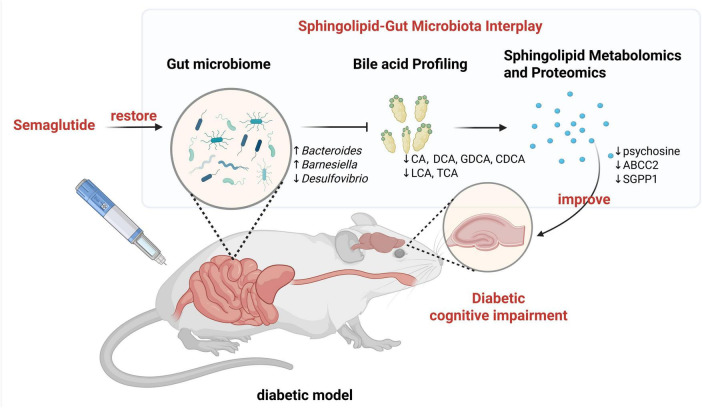
Proposed mechanism of semaglutide’s neuroprotective action in DCI. SE ameliorates diabetic cognitive impairment by targeting a novel “gut microbiota-bile acid-sphingolipid” axis. It restores a healthy gut microbiome, which normalizes bile acid profiles in the gut and brain. This, in turn, corrects pathogenic sphingolipid metabolism in the brain (e.g., by downregulating SGPP1), ultimately improving synaptic integrity and cognitive function. Created using BioRender.com.

In conclusion, our work uncovers a previously unrecognized “gut microbiota–bile acid–sphingolipid” axis as a critical contributor to the pathology of DCI (Figure 7). Our findings suggest that SE ameliorates neurodegeneration through a multifaceted mechanism, potentially involving both a peripherally-mediated restoration of the gut-brain axis and a more direct central action that converges on the regulation of sphingolipid homeostasis. While acknowledging the study’s exploratory nature, these findings not only deepen our understanding of DCI pathogenesis but also position the gut-brain axis as a prime therapeutic target for treating neurodegeneration in the context of metabolic disease.

## Data Availability

The datasets presented in this study can be found in the Zenodo repository with the following DOIs: Proteomics: https://doi.org/10.5281/zenodo.18299092, Metabolomics/Bile Acids: https://doi.org/10.5281/zenodo.18230322 (Version v2), and Shotgun Metagenomics: https://doi.org/10.5281/zenodo.18333340 and https://doi.org/10.5281/zenodo.18309443.
