# Supplementary material for: Multi-omics profiling implicates gut microbiota-sphingolipid interplay in the neuroprotective effects of semaglutide on diabetic cognitive impairment
Source: Front Microbiol. 2026 Mar 26;17:1705784. doi: 10.3389/fmicb.2026.1705784 (PMC13061865; doi:10.3389/fmicb.2026.1705784)

Supplementary Material

**Supplementary Table 1: Detailed Results of Analysis of Covariance (ANCOVA) for Key Variables.**

**Part A: Test of Inter-Subject Effects**

| **Dependent Variable** | **Source** | **df** | **F-value** | **p-value** |
| --- | --- | --- | --- | --- |
| Time in target quadrant | Group | 3 | 15.117 | <0.001 |
|  | Body Weight | 1 | 0.146 | 0.706 |
|  | Error | 26 |  |  |
| ABCA2 Expression | Group | 3 | 7.547 | 0.013 |
|  | Body Weight | 1 | 1.335 | 0.286 |
|  | Error | 7 |  |  |
| SGPP1 Expression | Group | 3 | 4.981 | 0.037 |
|  | Body Weight | 1 | 0.924 | 0.368 |
|  | Error | 7 |  |  |
| Psychosine | Group | 3 | 2.109 | 0.188 |
|  | Body Weight | 1 | 1.635 | 0.242 |
|  | Error | 7 |  |  |
| Cerebral GHDCA | Group | 3 | 7.249 | 0.015 |
|  | Body Weight | 1 | 0.723 | 0.423 |
|  | Error | 7 |  |  |
| Cerebral TCA | Group | 3 | 5.356 | 0.031 |
|  | Body Weight | 1 | 0.109 | 0.751 |
|  | Error | 7 |  |  |
| *Bacteroides* | Group | 3 | 7.908 | 0.004 |
|  | Body Weight | 1 | 1.287 | 0.281 |
|  | Error | 11 |  |  |

**Part B: Estimated Marginal Means (Post-Hoc Pairwise Comparisons, Bonferroni corrected)**

| **Dependent Variable** | **Comparison** | **p-value** |
| --- | --- | --- |
| Time in target quadrant | DM vs NC | <0.001 |
|  | DM+SE vs DM | <0.001 |
| ABCA2 Expression | DM vs NC | 0.121 |
|  | DM+SE vs DM | 0.015 |
| SGPP1 Expression | DM vs NC | 0.022 |
|  | DM+SE vs DM | 0.151 |
| Psychosine | DM vs NC | 0.493 |
|  | DM+SE vs DM | 0.188 |
| Cerebral GHDCA | DM vs NC | 0.031 |
|  | DM+SE vs DM | 0.106 |
| Cerebral TCA | DM vs NC | 0.047 |
|  | DM+SE vs DM | 0.120 |
| *Bacteroides* | DM vs NC | 0.007 |
|  | DM+SE vs DM | 0.075 |

**Supplementary Figure S1. Western blot validation of key sphingolipid-modulating proteins in the hippocampus and cortex.**

(A-D) Analysis of protein expression in the hippocampus. (A) Representative Western blot images showing the protein levels of ABCA2, SGPP1, CERS2, and the loading control GAPDH across all four experimental groups. (B-D) Densitometric quantification of the relative protein expression of (B) ABCA2, (C) SGPP1, and (D) CERS2, normalized to GAPDH.

(E-H) Analysis of protein expression in the cerebral cortex. (E) Representative Western blot images showing the protein levels of ABCA2, SGPP1, CERS2, and GAPDH. (F-H) Densitometric quantification of the relative protein expression of (F) ABCA2, (G) SGPP1, and (H) CERS2, normalized to GAPDH.

Data are presented as mean ± SD (n=3 mice per group). Statistical significance was determined by one-way ANOVA followed by Tukey's post-hoc test. **p* < 0.05, ***p* < 0.01, ****p* < 0.001. ns, not significant.

**Supplementary Figure S2. Uncropped and re-assembled original Western blot membranes related to Supplementary Figure S1.**

These figures display the original, uncropped chemiluminescence images of the Western blot membranes used for the quantification of ABCA2, SGPP1, CERS2, and the loading control GAPDH.

Note on image acquisition and presentation: To probe for multiple proteins (e.g., ABCA2, SGPP1, and GAPDH) from the same set of protein lysates run on a single gel, the full PVDF membrane was horizontally cut into strips according to the expected molecular weight ranges of the target proteins. Each strip was then incubated with its respective primary antibody and imaged separately. The images presented here are the digitally re-assembled views of these individual strips to show the alignment of the bands from the same original membrane. All lanes shown for a given blot image were run on the same gel. The positions of molecular weight markers (in kDa) are indicated, and the bands used for quantification are highlighted with red boxes.

**
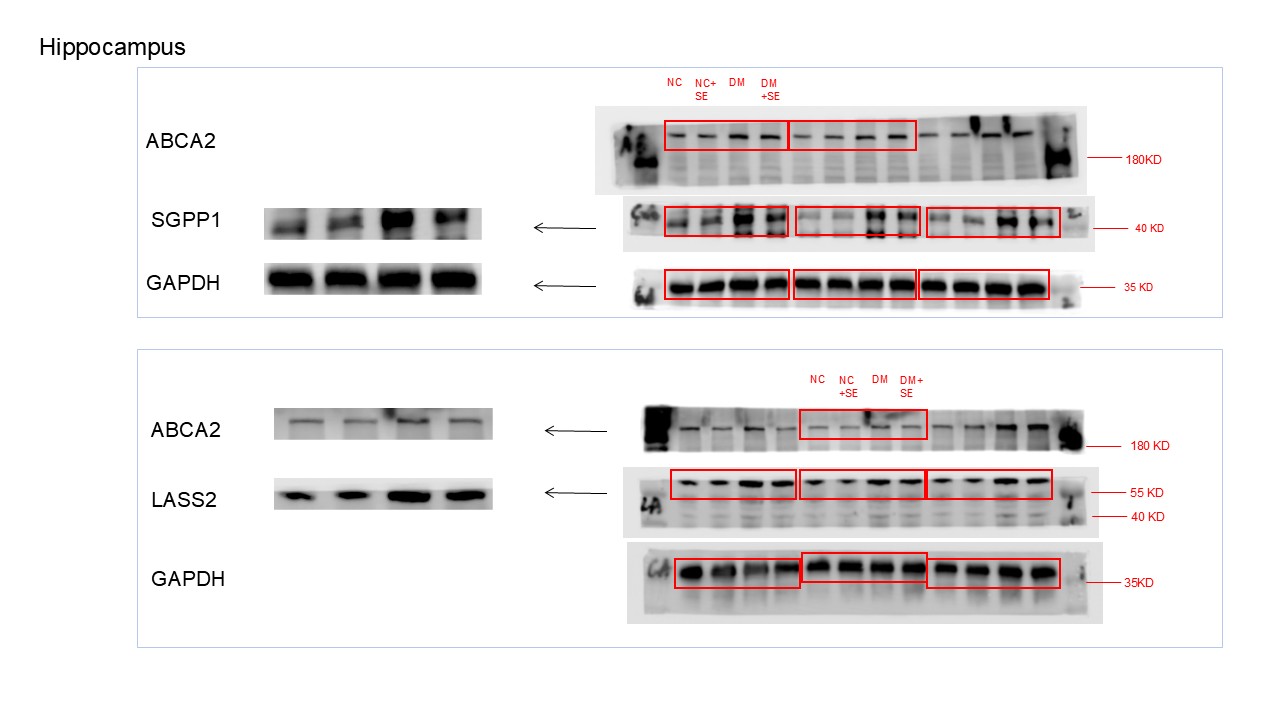
**
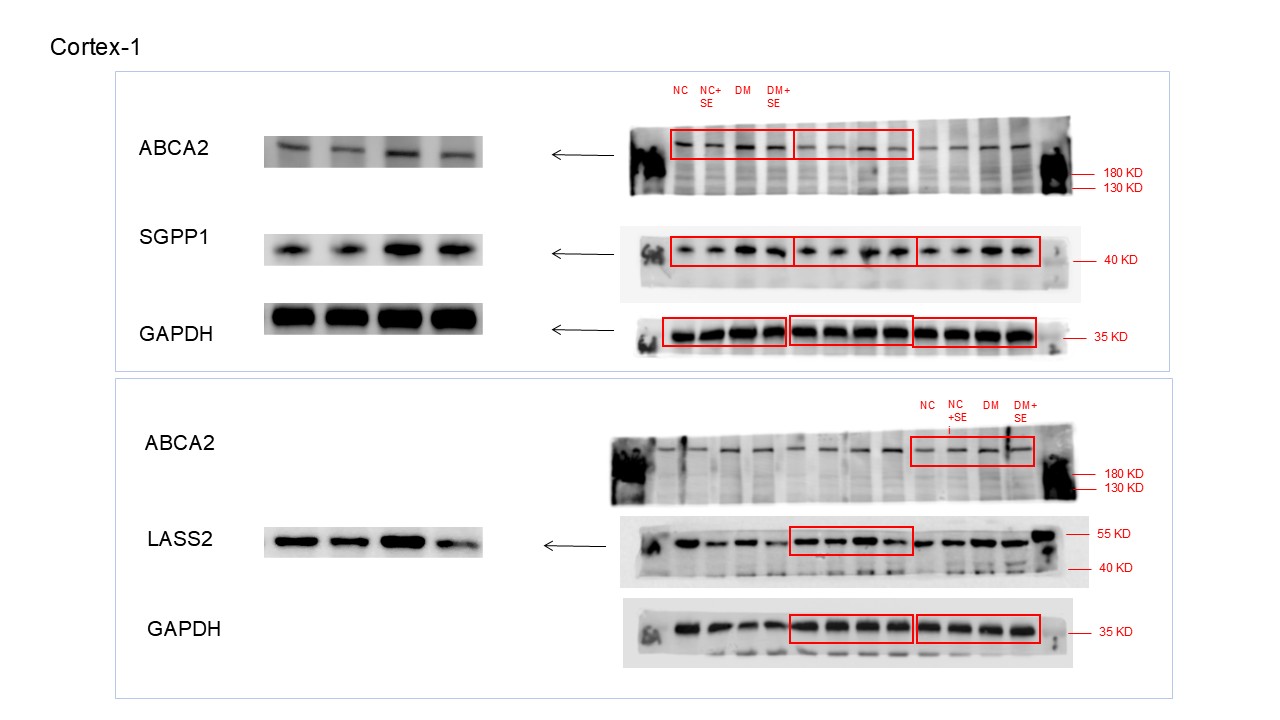

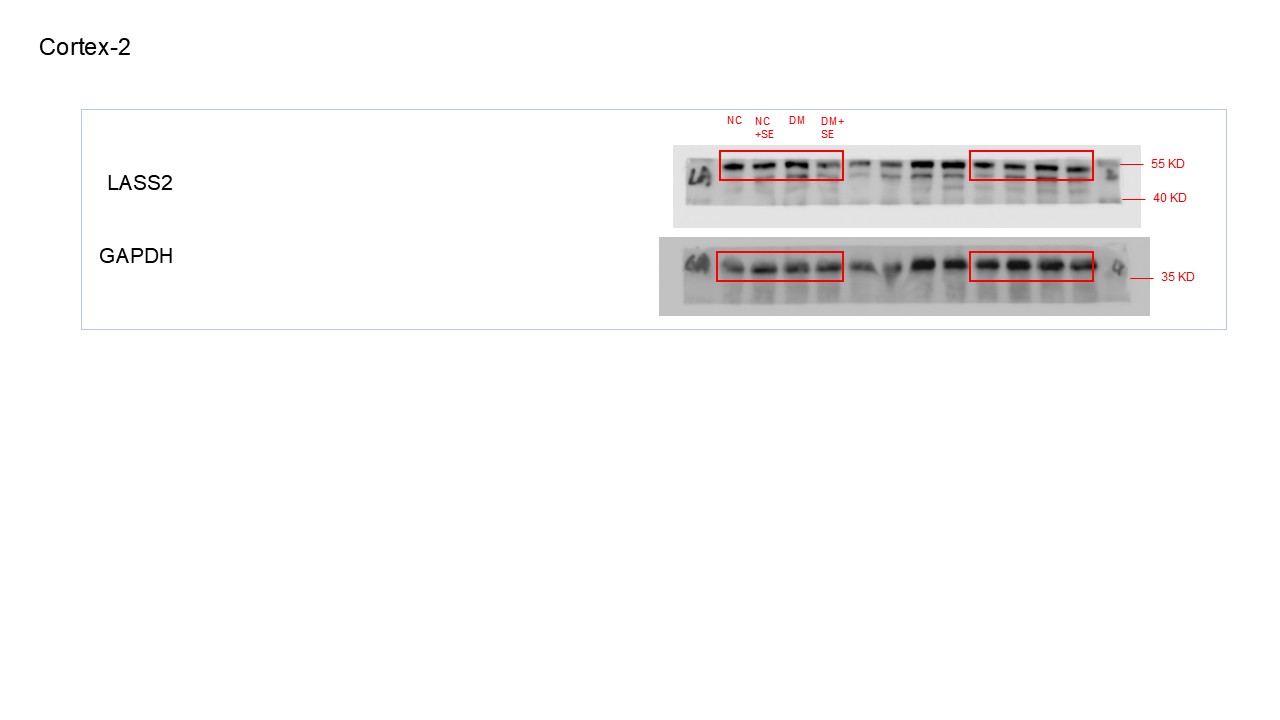

Supplement: Supplementary file 1 [file Table_1.docx]
