# Supplementary figures and images for: Multi-omics profiling implicates gut microbiota-sphingolipid interplay in the neuroprotective effects of semaglutide on diabetic cognitive impairment
Source: Front Microbiol. 2026 Mar 26;17:1705784. doi: 10.3389/fmicb.2026.1705784 (PMC13061865; doi:10.3389/fmicb.2026.1705784)

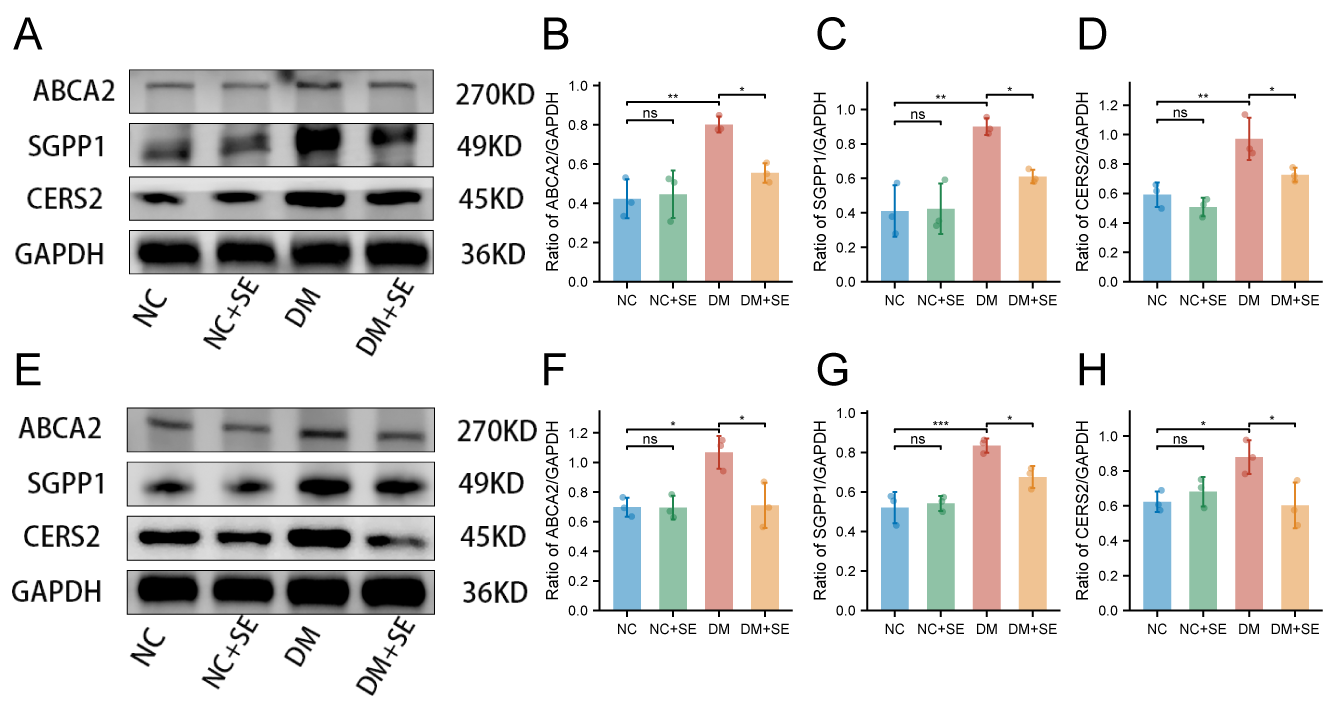

Supplement: Supplementary file 2 [file Image_1.tif]
